# Comparison of Teaching Methods in a Culinary Medicine Elective for Medical Students: In-Person Lectures Versus Inverted Classroom Model

**DOI:** 10.1177/23821205261422886

**Published:** 2026-03-07

**Authors:** Anna Manuela Plogmann, Selina Böttcher, Heidi Schwarzer, An Pham Trongan Ho, Anja Constien, Janna Gille, Karsten Weylandt, Uwe Neumann, Birgit Ellrott, Thomas Ellrott

**Affiliations:** 1 9175 Medical Centre, Justus-Liebig-University Gießen, Gießen, Germany; 2 Medical Department, Division of Gastroenterology, Oncology, Hematology, Rheumatology and Diabetes, ukrb, 477107 Brandenburg Medical School, Theodor Fontane, Neuruppin, Germany; 3 Institute for Nutrition and Psychology, 84922 Georg-August-University Göttingen Medical Centre, Göttingen, Germany; 4 Dietitian-School 14973 , University Hospital of Gießen and Marburg, Gießen, Germany; 5 834032 Culinary Medicine Germany e.V., Altenberge, Germany

**Keywords:** culinary medicine, inverted classroom, teaching kitchen, nutrition education for health professionals, interprofessional education

## Abstract

**Background:**

Since 2020, Culinary Medicine (CM) has been offered as an elective for medical students at several German universities. Recently, innovative teaching models such as the inverted classroom have been introduced. This study investigates whether the inverted classroom model, using online self-learning modules in the CM elective, improves students’ nutritional knowledge and counseling skills compared with traditional in-person lectures.

**Methods:**

The CM elective consisted of seven modules on dietary principles and disease-specific dietary therapy. The in-person cohort attended traditional lectures, while the inverted classroom cohort completed weekly online self-learning modules covering the same nutrition therapy content. Both groups participated in identical hands-on cooking sessions in teaching kitchens. Teaching effectiveness was assessed using standardized self-assessment questionnaires on counseling competencies, nutrition knowledge, eating habits, and mental well-being (WHO-5), administered before and after the elective. Paired and unpaired *t*-tests were used for analysis.

**Results:**

The study included 69 students in the inverted classroom and 80 in the in-person cohort. Both formats, when combined with hands-on cooking courses, led to significant improvements in nutritional knowledge, self-reported counseling skills, and practical abilities. No significant differences were observed between the cohorts.

**Conclusion:**

The inverted classroom model is as effective as in-person lectures when combined with hands-on cooking courses in a CM curriculum. Institutions can select the format based on local resources without compromising teaching outcomes. This study highlights the advantages and disadvantages of each method, supporting institutions in the development of improved CM curricula.

## Introduction

Lifestyle modifications, particularly in nutrition, impact significantly overall health.^1^ Dietary patterns are closely connected to the onset of illnesses including cardiovascular and metabolic disorders, cancer, and certain neurological diseases. Recognizing and implementing benefits of nutrition, not only for individuals but also for the public healthcare system, could improve global health significantly.^2^

It is therefore imperative to integrate nutrition education into the curriculum of medical students.^3^ A profound understanding of the impact of nutrition is essential to minimize knowledge gaps for healthcare specialists and their patients. Evidence-based knowledge needs to be the base when it comes to recommendations that align with patients’ lifestyles, preferences, and habits. However, nutrition education remains nonmandatory in medical schools in Germany and many other European countries.^4^ Considering the profound global and individual implications of nutrition, innovative approaches to nutritional education are urgently needed.^5^ Healthcare professionals including doctors, medical students, dietitians, and nurses should possess the expertise to provide evidence-based recommendations collaboratively across disciplines and effectively function as a cohesive team in nutrition-related domains.^6^

When it comes to the transfer of knowledge into practice, Culinary Medicine (CM) is a promising way of achieving this goal. CM is both a discipline and a teaching approach in clinical and public health education. It offers practical hands-on nutrition education and food-focused nutrition knowledge along with helpful cooking skills.^7^ The interdisciplinary use of teaching kitchens (TKs), where professionals, students or patients come together, enables participants to formulate recommendations for individuals in their everyday life.^8^ CM focuses on helping individuals make healthy dietary choices that support disease prevention, recovery, and improved quality of life. Its central aim is to empower patients to care for their health independently, effectively, and with satisfaction through food and beverages.^9^

The earliest cooking and nutrition elective in an American medical school was introduced in 2003.^10^ The first CM curriculum in Germany was implemented in Göttingen in 2020. The curriculum combines theoretical information about the principles of dietary management of various diet-related diseases and hands-on cooking courses where the corresponding dietary therapy is shown in practice. Since 2021 multicentered CM courses, based on the curriculum of the University of Göttingen, are also held at Brandenburg Medical School and the University of Gießen.^11^ CM had been taught in-person and virtually at these medical schools.^4^

Recent evidence underlines the substantial influence of teaching methods on student learning outcomes.^12^ Given the diversity of learning styles, a mismatch between instructional approaches and students’ learning preferences may impede educational outcomes. To address these differences, alternative learning strategies beyond the traditional lecture format should be considered to meet the diverse interests and abilities of a wider student population.^13^ The concept of the inverted classroom emerges from these findings: The inverted classroom refers to a model where activities traditionally carried out in class are shifted outside the classroom. Consequently, outside tasks are integrated into the classroom.^14^

The availability of digital networks and resources has enabled the development of new teaching models. The inverted classroom model comprises two parts: In the first part students can study freely using online learning platforms in a self-directed manner. The second part is performed in a classroom in an in-person setting to transfer and deepen the studied contents through peer group interactions. This approach shifts the emphasis from mere knowledge transmission to active information processing. Traditional lectures often lead to decreased attention and reduced concentration among students. In contrast, modern learning models leverage diverse resources such as short abstracts, videos, games, and images to sustain student engagement. Additionally, digital courses offer flexible, student-centered, and immersive learning opportunities by being accessible throughout the day without temporal constraints. The improvement of learning fields such as analyzing, synthesizing, and evaluating can lead to a higher cognitive learning process as students are taking part in in-person formats after individualized preparation. Moreover, peer collaboration and tutor guidance further reinforce these learning outcomes.^15^

The positive effects of practical CM training on medical students, such as improved dietary behaviors, greater nutrition knowledge, and increased confidence in counseling competencies have been well established.^7^ However, inverted classroom models are considered to improve learning, especially in clinical reasoning, by shifting information transmission to information processing.^15,16^

Starting in 2023, we implemented a new inverted classroom concept for the CM electives, which included digital self-learning modules. Students were asked to complete modules and additional tests independently using a digital platform and to acquire foundational knowledge. Afterward they took part in hands-on cooking courses in the TK to deepen their understanding.

The objective of this study was to evaluate the benefit of these online inverted classroom modules relative to in-person teaching lectures. This was assessed using a standardized comparative self-assessment questionnaire measuring counselling competencies, nutrition knowledge, eating habits, personal attitudes toward nutrition counselling in medical practice, and mental well-being (WHO-5).^11^

## Materials and Methods

### Design

The study was conducted at three medical schools in Germany (Göttingen, Gießen and Brandenburg). Two study cohorts were enrolled: The in-person cohort studied theory in a face-to-face lecture directly before the hands-on cooking class in the TK and the inverted classroom cohort completed theoretical content in advance through online self-learning modules. The in-person cohort was drawn from all three universities, whereas the inverted classroom cohort was implemented only at Göttingen. Cohorts were not randomized, as the inverted classroom elective represented a further development of the classic CM electives and was compared to in-person teaching courses performed in previous years. The data from the virtual cohort were derived from the same cohort from Böttcher et al (2023).^11^

### Participants

The study involved medical students who had selected CM as an elective course. Only those who regularly attended the elective sessions were included, while students with irregular attendance or incomplete assessments were excluded. Participants were medical students who had chosen CM as an elective course. Inclusion criteria were frequent participation in the electives. Students with infrequent participation or who did not complete the assessments were excluded from the study.^11^

### CM Elective with Initial In-Person Lectures

In 2022 and 2023, the in-person CM electives were conducted at the 3 participating universities. These elective courses included 28 lecture hours (45 min. each) and were divided into 2 parts: The first part consisted of a 45-min initial lecture about nutrition therapy for patients with diet-related diseases. The second part was a practical learning experience in the TK for approximately 3 h. The curriculum covered seven key topics: healthy diet, malnutrition, dietary therapy of cardiovascular and metabolic disease's parts I and II, dietary management of gastrointestinal diseases, dietary therapy of kidney diseases, and dietary therapy of inflammatory rheumatic, orthopedic, neurological, and pulmonary diseases. These were studied either in 7 modules over 7 weeks (4 lecture hours per day) or as a block of 4 to 5 days (5 to 7 lecture hours per day).^11^

The interprofessional team of the CM elective consisted of nutritionists, dietitians, physicians, cooks trained in dietetics and tutors (medical students who previously completed the CM curriculum). The CM electives were structured according to the German consensus paper “*Manual of nutritional therapy in patient care”* (LEKuP).^17^ Further details of this approach are described in Böttcher et al (2023).^11^

### Inverted Classroom Model

To enhance students’ learning conditions, the CM elective was redesigned in 2023 following an inverted classroom approach by shifting lectures to an online platform. The learning management system *Integrated Learning, Information and Work Cooperation System* (ILIAS) was implemented for this purpose. The teaching materials from the traditional lectures (as described in the culinary medicine elective with initial in-person lectures section) were adapted and transcribed to the online platform, maintaining the same seven subject areas of the traditional CM elective. Instead of attending lectures directly before practical courses in the TK, students were asked to complete digital modules in a self-directed manner in a time frame of 5 to 7 days before participating in the hands-on cooking courses.

Preparation of appropriate photos and videos was realized in the TK in Göttingen. Material was transformed into teaching sessions that included questions, quizzes, or video tutorials (see Appendices 1 and 2 in Supplemental Material S1).

Quiz-based learning is a highly effective instructional strategy that leverages the principles of active recall, spaced repetition, and formative assessment.^18^ Questions and quizzes enhance learning through immediate feedback and represent an effective method for promoting long-term retention of knowledge^18,19^ Active learning strategies embedded within video-based education substantially improve learners’ retention, comprehension, and transfer of knowledge. The incorporation of interactive elements, such as embedded quizzes and reflective tasks, has been shown to enhance learner motivation, understanding, and memory performance.^20^ Moreover, the ability to flexibly navigate video content supports self-paced learning among students.^21^

Each module contained brief written sections and included links to external sources or websites for further study. To ensure that students had engaged with the theoretical content before participating in the practical sessions in the TK, each module concluded with a final test consisting of 10 questions. The total estimated time per module ranged from 45 to 60 min, making it comparable in duration of the previous in-person lectures. Specifically, the self-learning modules were designed to require approximately 30 to 45 min for completion, followed by an estimated 15 min to complete the test.

Both course formats required approximately 29 h in total. Students in the inverted classroom cohort spent less time completing the tests overall than on preparing case -reports. In the TK, more time was available. However, this also included a brief thematic introduction, resulting in a total duration that is nearly identical to that of the in-person cohort.

Table 1 presents the components implemented in each instructional format: the inverted classroom cohort and the in-person cohort.

**Table 1.** Comparison of Components—Inverted Classroom and in-Person Cohort.

Components	Inverted-Classroom	In-Person
Seven in-person lectures	-	X
Preparation of 1 case-report	-	X
Seven self-learning modules	X	-
Test at the end of each self-learning module	X	-
Hands-on cooking class in teaching kitchens	X	X
Professional discussion of dietary and culinary aspects during dinner	X	X

### Evaluation Tools

To assess teaching effectiveness in both cohorts before and after the elective courses, a standardized comparative self-assessment questionnaire was administered online via LimeSurvey (see Supplemental Material S2). The questionnaire covered self-reported counseling competencies, personal attitudes toward nutrition counseling in medical practice, nutrition knowledge, mental well-being (measured with the WHO-5) and eating habits.^11^

Items regarding counseling competencies and personal attitudes towards nutrition counseling were adapted from Razavi et al (2020).^22^ The questionnaire included 25 different counseling topics related to various diets and nutrition principles for diseases. Some adaptations were necessary. As an example: Given the limited awareness of the DASH Diet in Germany, dietary recommendations for patients with arterial hypertension from LEKuP^17^ were used instead.

Nutrition knowledge was evaluated with16 multiple-choice questions pertaining to dietary therapy for various diet-related diseases according to LEKuP guidelines. Mental well-being was assessed using the WHO-5 well-being index. A 5-point Likert scale was used to measure eating habits, including the frequency of consumption per week across 12 food groups, aligning with the official food-based dietary guidelines for healthy eating and drinking published by the German Nutrition Society (DGE).^23^

Participation in the evaluation was voluntary, with the presurvey being conducted up to 2 weeks prior to the course start date. The postsurvey was administered within 2 weeks following the end of the course. The evaluation followed the same procedure as in previous studies by our CM team.^11^

### Data Collection and Analysis

Data were collected using LimeSurvey (Version 3.24.2 + 201020), a free and open-source online survey platform written in PHP and distributed under the GNU General Public License. Participants generated individual pseudonyms at the beginning of the survey, which were used to link pre- and postsurvey responses anonymously. The data collected were exported to Microsoft Excel (Version 16.75.2) and analyzed using IBM SPSS Statistics (Version 28.0.1.0).

Normal distribution of the data was assessed using quantile-quantile plots. Paired *t*-tests were used to compare pre- and postsurvey results within both the inverted classroom and in-person cohorts. Comparisons between the 2 cohorts were performed with unpaired *t*-tests for the questionnaire sections on counseling competencies, eating habits, and the WHO-5 well-being index. Statistical significance was set at *P* < .05. Effect sizes were calculated using Cohen's d, with the following interpretation: no effect for *d* < 0.2, small effect for *d* = 0.2-0.49, medium effect for *d* = 0.5-0.79, and large effect for *d* ≥ 0.8.

All questionnaire items were treated as interval-scaled data using a Likert scale. Graphs and statistical calculations were performed in IBM SPSS Statistics.

The reporting of this study conforms to the STOBE statement (cohort studies) (see Supplemental Material S3).

### Ethics Approval

The ethics committee of the University of Göttingen Medical Centre approved the use of data from teaching evaluations for scientific purposes on September 23, 2022 under the application number 25/9/22.

## Results

### Participants

The inverted classroom cohort included 105 medical students who completed the CM curriculum at the UMG in the summer semester of 2023 (*n* = 49) and the winter semester of 2023/2024 (*n* = 56). Complete datasets from the voluntary pre- and postsurveys were available for 69 medical students (65.7%) and included in the statistical analysis. The previous in-person cohort consisted of 149 medical students who completed the CM curriculum, with 80 complete voluntary pre- and postsurveys included in the analysis (53.6%). A detailed overview of student characteristics is presented in Table 2.

**Table 2.** Baseline Demographics of Participants.

Participant Demographics	Inverted Classroom	In-Person	*P*-Value
Mean age (years ± SD)	24.35 (± 4.28)	24.5 (±6.53)	.867*
Female/male/other (*n*)	49/20/-	57/23/ -	0,975/0,975/-**
Mean semester (± SD)	6.92 (± 1.33)	6.26 (±4.26)	.192*
Completed professional training before starting medical studies (*n*)	13	31	.013**
Completed academic degree before starting medical studies (*n*)	3	8	.317**
City: Göttingen (*n*)	69 (100%)	22 (40.1%)	<.001**
City: Gießen (*n*)	-	32 (27.5%)	<.001**
City: Brandenburg (*n*)	-	26 (32.5%)	<.001**
*N* (total)	69	80	

SD, standard deviation; *t-test, ** chi-square test.

All participants were medical students who had chosen CM as their mandatory elective course during either the clinical or preclinical phase of their studies, selected from an elective catalog provided by their respective universities. To evaluate statistical differences between the 2 cohorts, unpaired *t*-tests and chi-square tests were conducted.

### Counselling Competencies

The CM courses demonstrated significantly positive effects across all 25 categories of counseling competencies in both cohorts (inverted classroom: *P* < .001; in-person: *P* < .001) (Table 3). Unpaired *t*-tests revealed significant differences between the cohorts in 2 categories: celiac disease (*P* = .043) and dietary fiber (*P* = .012) (see Tables S1 and S2 in Supplemental Material S1). Overall, the in-person cohort showed partially greater changes in mean values (inverted classroom: 0.54-1.43; in-person: 0.7-1.7).

**Table 3.** Mean Changes in Counselling Competencies.

Topic	Before Inverted Classroom *M* (*SD*)	After Inverted Classroom *M* (*SD*)	Before In-Person *M* (*SD*)	After In-Person *M* (*SD*)	Inverted Classroom *MD* (*SD*)	In-Person *MD* (*SD*)
Mediterranean diet	3.07 (1.15)	3.93 (1.02)	3.18 (1.15)	4.30 (0.64)	0.85 (1.09)*	1.13 (1.04)*
Arterial hypertension diet	3.10 (1.20)	4.19 (0.91)	3.26 (1.09)	4.36 (0.57)	1.09 (1.22)*	1.10 (1.16)*
Vegetarian diet	3.22 (1.11)	4.09 (0.98)	3.54 (1.09)	4.34 (0.71)	0.87 (1.18)*	0.80 (0.96)*
Low-fat diet	3.24 (1.00)	4.07 (0.93)	3.43 (0.99)	4.19 (0.73)	0.84 (0.96)*	0.76 (1.02)*
High-protein diet	3.28 (1.13)	4.19 (0.93)	3.23 (1.06)	4.18 (0.67)	0.91 (1.08)*	0.95 (1.12)*
Serving size	3.00 (1.21)	3.81 (1.02)	2.90 (1.15)	3.95 (0.72)	0.81 (1.16)*	1.05 (1.07)*
Moderate alcohol	3.60 (1.18)	4.19 (0.99)	3.70 (1.07)	4.44 (0.65)	0.60 (1.26)*	0.73 (1.00)*
Eating disorders	3.40 (1.16)	3.94 (1.11)	3.40 (1.05)	4.10 (0.73)	0.54 (1.09)*	0.70 (1.04)*
Cholesterol	3.33 (1.15)	4.13 (0.94)	3.19 (1.08)	3.94 (0.73)	0.81 (1.18)*	0.75 (1.02)*
Diabetes diet	3.25 (1.39)	4.37 (0.80)	3.04 (1.16)	4.21 (0.61)	1.12 (1.41)*	1.17 (1.14)*
Diabetes weight loss	3.46 (1.35)	4.37 (0.85)	3.29 (1.13)	4.36 (0.68)	0.91 (1.23)*	1.07 (1.45)*
Obesity weight loss	3.24 (1.29)	4.22 (1.00)	3.23 (1.18)	4.43 (0.63)	0.99 (1.33)*	1.20 (1.22)*
Omega fats ω-3 and -6	2.78 (1.20)	3.88 (1.04)	2.56 (1.15)	3.80 (0.84)	1.10 (1.24)*	1.23 (1.08)*
Dietary fats	2.85 (1.15)	3.79 (0.99)	2.65 (1.13)	3.83 (0.79)	0.94 (1.14)*	1.17 (1.22)*
Antioxidants	2.63 (1.13)	3.67 (0.94)	2.60 (1.15)	3.55 (0.89)	1.04 (1.09)*	0.95 (1.20)*
Calories	3.00 (1.24)	3.97 (1.06)	2.96 (1.14)	3.93 (0.89)	0.97 (1.41)*	0.96 (1.10)*
Hydration	3.60 (1.22)	4.40 (0.92)	3.70 (1.01)	4.43 (0.65)	0.81 (1.10)*	0.68 (1.10)*
Celiac disease	2.67 (1.27)	3.94 (1.04)	2.49 (1.28)	4.19 (0.79)	1.27 (1.20)*	1.70 (1.33)*
Food allergies	2.40 (1.27)	3.84 (0.93)	2.26 (1.12)	3.94 (0.78)	1.43 (1.32)*	1.67 (1.20)*
Glycemic index	2.51 (1.22)	3.61 (1.13)	2.24 (1.16)	3.65 (0.87)	1.10 (1.30)*	1.42 (1.20)*
Fiber	3.21 (1.25)	4.09 (0.92)	2.90 (1.18)	4.29 (0.71)	0.88 (1.24)*	1.38 (1.18)*
Food label	2.88 (1.26)	3.94 (1.00)	2.79 (1.33)	4.16 (0.70)	1.06 (1.43)*	1.37 (1.25)*
Osteoporosis	2.66 (1.34)	3.75 (1.08)	2.44 (1.27)	3.89 (0.74)	1.09 (1.37)*	1.45 (1.32)*
BMI	3.40 (1.18)	4.16 (0.99)	3.60 (0.97)	4.55 (0.59)	0.76 (1.07)*	0.95 (0.98)*
Aerobic exercise	3.72 (1.28)	4.30 (0.94)	4.53 (1.10)	4.25 (0.75)	0.85 (1.09)*	0.72 (1.09)*
**Overall**	**3.14** (**0.32)**	**4.03** (**0.21)**	**3.08** (**1.17)**	**4.14** (**0.72)**	0.95 (0.23)*	1.05 (1.13)*

M, mean; MD, mean difference (*P ≤ .001), see Tables S1 and S2 in Supplemental Material S1.

### Attitudes Toward Nutrition Counselling in Medical Practice

A significant positive change in personal attitudes toward nutritional counseling in medical practice was observed in both cohorts for almost all statements (Table 4): “*Counseling on nutritional issues should be part of every medical consultation, just like therapy and diagnosis”* (inverted classroom: *P* < .001; in-person: *P* = .036), “*Specific recommendations for changing eating behavior can help patients improve their eating habits”* (inverted classroom: *P* = .002; in-person: *P* = .006), and “*Physicians can influence their patients’ eating habits if they take the time to discuss the issue with them”* (inverted classroom: *P* < .001; in-person: *P* < .001). The average difference was greater in the in-person cohort (inverted classroom: −0.07-0.12; in-person: 0.22-0.36), but the differences between the two cohorts were not significant (see Tables S3 and S4 in Supplemental Material S1).

**Table 4.** Mean Changes in Attitudes Toward Nutrition Counselling in Medical Practice.

Question	Before Inverted Classroom *M* (*SD*)	After Inverted Classroom *M* (*SD*)	Inverted Classroom *MD* (SD)	Before In-Person *M* (*SD*)	After In-Person *M* (*SD*)	In-Person *MD* (*SD*)
Nutrition counselling should be routine	4.28 (0.87)	4.36 (0.83)	0.07 (0.80)*	4.36 (0.82)	4.59 (0.65)	0.22 (0.94)
Specific counselling can improve patients’ diets	4.51 (0.68)	4.63 (0.76)	0.12 (0.81)	4.61 (0.72)	4.84 (0.37)	0.22 (0.71)
Physicians’ counselling can improve patients’ diets	4.42 (0.80)	4.34 (0.83)	−0.07 (0.82)*	4.38 (0.83)	4.74 (0.54)	0.36 (0.76)*

M, mean; MD, mean difference (*P ≤ .001), see Table S4 in Supplemental Material S1.

Students in the inverted classroom cohort rated the third statement, “*Physician counseling can improve patients’ diets,”* significantly lower after the CM elective (MD before: 4.42; MD after: 4.34).

### Nutritional Knowledge

The presurvey results indicate that students in the inverted classroom cohort increased their nutritional knowledge by 19.2%, while the in-person cohort demonstrated a comparable knowledge gain of 18.9% (Table 5).

**Table 5.** Mean Changes in Nutrition Knowledge.

	M of Right Answering Participants Before Inverted Classroom (%)	M of Right Answering Participants After Inverted Classroom (%)	M of Right Answering Participants Before In-Person (%)	M of Right Answering Participants After In-Person (%)	Inverted Classroom MD	In-Person MD
1. Recommended diet form	79.0	99.0	71.3	92.5	19.4*	21.3*
2. Carbohydrate percentage	42.0	60.0	38.8	61.3	17.9	22.5
3. Salt	28.0	90.0	25.0	61.3	61.2*	36.3*
4. Free sugars	48.0	67.0	56.3	76.3	19.4	20.0
5. Recommended protein	63.0	87.0	48.8	85.0	23.9	36.2*
6. Malnutrition syndrome	51.0	97.0*	65.0	85.0	46.3*	20.0*
7. Therapy obesity	82.0	90.0	68.8	86.3	7.5	17.5
8. Gout	79.0	76.0	67.5	66.3	−3.0	−1.2
9. Monosaccharide gout	32.0	41.0	25.0	51.2	9.1	26.2*
10. Dyslipoproteinemia	18.0	26.0	26.3	28.7	7.6	2.4
11. Cereals for celiac disease	53.0	70.0	55.4	73.8	16.7	18.4
12. Chronic kidney disease therapy	47.0	70.0	56.3	61.3	22.7	5.0
13. Calcium oxalate stones	22.0	45.0	17.5	51.2	22.4	33.7*
14. Fructose malabsorption	45.0	84.0*	37.5	53.8	38.8 *	16.3
15. Omega-3 fatty acid	45.0	69.0	46.3	72.5	23.9	26.2*
16. Calcium and Vitamin D	24.0	24.0	33.8	36.3	0.0	2.5
**Overall**	**46**.**7**	**68**.**3**	**46**.**2**	**65**.**2**	**19**.**2**	**18**.**9**

M, mean; MD, mean difference (*P ≤ .001); see  Table S5 in Supplemental Material S1.

Significant differences between the two cohorts were observed in four specific questions (see Table S6 in Supplemental Material S1). The inverted classroom cohort achieved significantly better results in questions related to salt (*P* = .007), malnutrition (*P* = .001), and fructose malabsorption (*P* = .015). Conversely, the in-person cohort scored significantly higher on the question concerning gout (*P* = .004).

The inverted classroom cohort significantly improved their responses in 11 out of 16 questions. While students also demonstrated better results in questions 7, 9, 10, and 16, these changes were not statistically significant. The in-person cohort showed significantly better results in 12 out of 16 questions. Questions 8, 10, 12, and 16 were answered better, but these improvements were not statistically significant.

Both the inverted classroom and the in-person cohorts showed a decline in their results for question eight, which addressed “gout.” However, this decrease was not significant (see  Table S5 and S6 in Supplemental Material S1).

## Discussion

The inverted classroom model has emerged as a promising and innovative approach to enhancing student performance.^24^ This study represents the first implementation of an inverted classroom model utilizing online self-learning models in a CM elective in Germany. Students in the inverted classroom model achieved results comparable to those in the traditional in-person teaching format. The cohorts did not differ significantly with respect to counseling competencies, attitudes toward nutritional counseling in medical practice, and nutritional knowledge. Both teaching models demonstrated similar effects on knowledge gain and the development of competencies in nutritional medicine education.

The inverted classroom method offers several advantages, notably increased flexibility through digital self-learning modules.^25^ Additionally, the model supports interactive learning through interactive tests, quizzes, and interactive videos, which can enhance student engagement and motivation. The approach also fosters self-directed and immersive learning, critical skills in medical education.^26^

In other medical disciplines the inverted classroom model has already been shown to successfully enhance learning outcomes and practical skills. For example, Dahbi et al (2024) demonstrated significant benefits in knowledge transfer in oncology, highlighting the model's capacity to foster active learning, critical thinking, and practical skills, ultimately contributing to improved patient care.^27^ Similarly, Ganfornina et al (2023) reported improved exam results, increased motivation, and deeper understanding among students in neurophysiology courses using this approach.^28^ The integration of an inverted classroom model within a competency-based curriculum in psychiatry and psychotherapy likewise resulted in improved practical skills among medical students.^29^ The results of our study are consistent with these findings, indicating a broad potential and high acceptance of the inverted classroom model across different areas.

Nevertheless, the inverted classroom model entails certain challenges. Technical barriers can hinder its implementation, and the development and maintenance of self-learning modules can be resource-intensive and technically demanding. The model's effectiveness depends strongly on the quality of the provided materials and the availability of technical resources.^26,28^ Furthermore, it is essential to train instructors in the use of digital tools as well as to ensure their motivation and commitment to managing the online components.^30^ In our approach, the ILIAS platform was supported by technical staff and trained tutors who had previously completed the CM elective, which facilitated resolution of technical issues.

Moreover, the effectiveness of the inverted classroom model may vary depending on students’ individual skills and learning preferences. Students with strong time management abilities may benefit more from the flexibility of the model, whereas others may face challenges with self-organization.^28^ Likewise, learning styles can influence outcomes. While students with auditory and kinesthetic learning preferences may favor traditional teaching methods, those with visual and self-directed learning styles tend to respond better to the inverted classroom approach.^26^ To ensure adequate preparation and training success, students were asked to complete a mandatory online test prior to participating in the hands-on cooking class in the TK.

Unlike the undirected nature of blended learning, the inverted classroom model follows a structured format. Here the theory preparation occurs before in-person sessions, which are then used for deepening understanding and practical application. Bruijn-Smolders and Prinsen (2024) highlight the inverted classroom model as an effective method for content delivery, emphasizing the role of online learning communities, playful learning, and variety. In our study, these elements were incorporated through quizzes, interactive components, and practical cooking tips for daily life within the online self-learning modules, which likely enhanced students’ motivation and engagement. Although improvements in counseling competencies were observed, it remains unclear how much blended learning and inverted classroom models enhance social interaction and engagement. Further research is needed to explore this question.^31^

Test-based learning is another evidence-based method integrated into the online CM self-learning modules in this study. Regular retrieval exercises strengthen memory and facilitate long-term retention and knowledge transfer.^32^ Easily accessible and low-stress tests were used to motivate students and reduce exam-related anxiety.^33^ Feedback mechanisms were incorporated to enhance learning by providing students with specific, precise corrections to reinforce understanding.^34^ Although tests serve to validate module completion and reinforce learning, it remains challenging to ensure that students do not use search engines or artificial intelligence tools for assistance. Nevertheless, the use of such tools for web-based information retrieval may also enhance learning.^35^ In our questionnaires, we explicitly prohibited such assistance, but its usage cannot be definitively verified or prevented.

The review by Tan et al (2022) highlights the diversity and inconsistency of teaching methods employed in CM to convey nutritional knowledge and counseling skills.^36^ While both the inverted classroom model and traditional in-person courses in this study improved students’ competencies and knowledge, it remains unclear which approach is more effective overall. The inverted classroom model, with its flexibility and capacity for personalized learning, offers a valuable alternative or complement to traditional in-person instruction. The findings indicate that both methods are beneficial, however, further research is needed to develop standardized course recommendations and to mandatorily integrate CM concepts into medical curricula. Such integration would emphasize the significance of nutrition and nutritional medicine for medical students. Continued research and development are essential to optimize these teaching methods and establish a standardized framework, thereby fully realizing their potential in CM education.

CM electives are increasingly being introduced at German medical schools with the aim of training future doctors in evidence-based nutrition. The integration of practical and theoretical elements in CM promotes the development of comprehensive competencies essential for both individual patient care and public health.^22^ Emphasizing lifestyle-related skills and the role of preventive measures underscores the need for CM's integration into medical education. This approach provides long-term benefits not only for medical students but also for patients and the healthcare system, contributing to more effective and holistic strategies for disease prevention and management.^2,22^

The development and expansion of CM courses urgently require standardized curricula, best practices, and assessment methods. These measures are essential not only for quality assurance but also for promoting sustainable and inclusive public health.^3^ As the burden of chronic diseases grows, integrating CM into medical curricula becomes increasingly important, as future physicians must manage these challenges through prevention and lifestyle interventions.^37^ Establishing and implementing standardized best practices in nutrition education are critical to prepare medical students for these modern healthcare challenges.

The consensus statement by Eisenberg et al (2024) provides recommended nutrition competencies for medical students and physicians, aiming to establish a unified foundation for nutrition education.^38^ It emphasizes that nutrition should be effectively integrated into training and addressed in disease prevention, diagnosis, and therapy. The practical application of nutritional knowledge is highlighted as central, focusing on patient-centered education. Translating theoretical knowledge into everyday life behaviors is key to supporting sustainable lifestyle changes.^38^

Table 6 provides a comparative overview of the advantages and disadvantages of the inverted classroom and in-person teaching formats, detailing the workload related to main requirements.

**Table 6.** Comparison of the Main Requirements in the Teaching Formats.

Requirements for	Inverted Classroom	In-Person
Programming, photography and filming for self-learning modules	Very high	None
Programming tests at the end of each module	High	None
Conducting self-learning modules	Low	None
Conducting and evaluating tests at the end of each module	Low	None
Preparation of in-person lectures	None	High
Preparation of case-reports	None	High
Conducting of in-person lectures	None	Medium-high
Conducting and evaluating case-reports	None	Medium-high
Maintenance and updates	Low	Low

Our findings contribute to a deeper understanding of effective teaching methods, aiding the development of standardized CM curricula for medical students. The results demonstrate that the inverted classroom model achieves comparable outcomes in nutritional knowledge and counseling competencies when compared to in-person theoretical teaching. Since both teaching formats have distinctive advantages and disadvantages (Table 6), institutions can select the approach that best aligns with their available resources without compromising teaching success. While the initial requirements for developing the self-learning modules and tests in the inverted classroom model (including filming and photography) are substantial, the subsequent workload for course delivery and evaluation is much lower compared to in-person lectures and case-reports. However, the initial barriers to launching a new CM elective are generally lower with the in-person lectures and case-reports.

### Limitations of the Study

The relatively small sample sizes of both the in-person and inverted classroom cohorts, as well as the lack of an a priori sample size calculation, represent limitations of this study, although statistical significance was still achieved. Variations in teaching teams and resource availability across the 3 study sites may have influenced outcomes despite use of identical teaching materials. Furthermore, as this study was not randomized, our results may be more vulnerable when drawing general conclusions. The reliance on self-reported data introduces potential bias, as subjective assessments may reduce objectivity and increase interindividual variability.

The in-person cohort included a significantly higher proportion of students with prior professional training, which may have influenced learning outcomes independently of the teaching method. Additionally, only a subset of all students participating in the CM courses completed the evaluations, which likely introduces selection bias, as the analyzed sample may overrepresent more motivated students, thereby limiting generalizability. It is plausible that more motivated and proficient students were more likely to participate in both surveys.

Direct individual comparisons between the in-person and inverted classroom cohorts were not feasible due to differences in course registration procedures.

Despite these limitations, the findings underscore the comparable effectiveness of both teaching methods in enhancing nutritional knowledge and counseling competencies. Moreover, the study highlights the respective advantages and disadvantages of each approach, providing valuable guidance for institutions implementing new CM curricula to improve in medical education.

## Conclusion

The integration of CM through the inverted classroom model presents a promising strategy to enhance medical nutrition education and improve practical patient care. By combining theoretical self-study with interactive hands-on sessions, this approach offers a flexible and adaptable framework well-suited for university settings. Due to its advantages, the inverted classroom model may emerge as the preferred teaching method for CM in medical curricula.

Optimal CM education requires careful planning and the establishment of interdisciplinary teams, including dedicated technical support to facilitate online learning. Whenever CM is integrated into medical contexts, available resources as well as institutional and learner-specific needs must be considered.

While comprehensive resources can enhance the learning experience, studies have demonstrated that even low-threshold approaches, such as fully virtual cooking courses,^7,11,39^ can lead to significant improvements in dietary habits and culinary skills. Therefore, the emphasis should be on developing feasible programs tailored to the specific needs and contexts of each educational setting.

A shift in didactic planning, particularly through the increased use of digital platforms and their interactive capabilities, opens new possibilities. These developments will be further supported and expanded through ongoing research, particularly with the responsible and mindful integration of artificial intelligence. This study supports the role of the inverted classroom model as a central element of CM courses, situated within the broader context of digital transformation in education and suggests its consideration in future curriculum revisions in medical education.

## Supplemental Material

sj-docx-1-mde-10.1177_23821205261422886 - Supplemental material for Comparison of Teaching Methods in a Culinary Medicine Elective for Medical Students: In-Person Lectures Versus Inverted Classroom Model Supplemental material, sj-docx-1-mde-10.1177_23821205261422886 for Comparison of Teaching Methods in a Culinary Medicine Elective for Medical Students: In-Person Lectures Versus Inverted Classroom Model by Anna Manuela Plogmann, Selina Böttcher, Heidi Schwarzer, An Pham Trongan Ho, Anja Constien, Janna Gille, Karsten Weylandt, Uwe Neumann, Birgit Ellrott and Thomas Ellrott in Journal of Medical Education and Curricular Development

sj-pdf-2-mde-10.1177_23821205261422886 - Supplemental material for Comparison of Teaching Methods in a Culinary Medicine Elective for Medical Students: In-Person Lectures Versus Inverted Classroom Model Supplemental material, sj-pdf-2-mde-10.1177_23821205261422886 for Comparison of Teaching Methods in a Culinary Medicine Elective for Medical Students: In-Person Lectures Versus Inverted Classroom Model by Anna Manuela Plogmann, Selina Böttcher, Heidi Schwarzer, An Pham Trongan Ho, Anja Constien, Janna Gille, Karsten Weylandt, Uwe Neumann, Birgit Ellrott and Thomas Ellrott in Journal of Medical Education and Curricular Development

sj-pdf-3-mde-10.1177_23821205261422886 - Supplemental material for Comparison of Teaching Methods in a Culinary Medicine Elective for Medical Students: In-Person Lectures Versus Inverted Classroom Model Supplemental material, sj-pdf-3-mde-10.1177_23821205261422886 for Comparison of Teaching Methods in a Culinary Medicine Elective for Medical Students: In-Person Lectures Versus Inverted Classroom Model by Anna Manuela Plogmann, Selina Böttcher, Heidi Schwarzer, An Pham Trongan Ho, Anja Constien, Janna Gille, Karsten Weylandt, Uwe Neumann, Birgit Ellrott and Thomas Ellrott in Journal of Medical Education and Curricular Development
