# Supplementary material for: Comparison of Teaching Methods in a Culinary Medicine Elective for Medical Students: In-Person Lectures Versus Inverted Classroom Model
Source: J Med Educ Curric Dev. 2026 Mar 7;13:23821205261422886. doi: 10.1177/23821205261422886 (PMC12967350; doi:10.1177/23821205261422886)
Supplement: sj-docx-1-mde-10.1177_23821205261422886 - Supplemental material for Comparison of Teaching Methods in a Culinary Medicine Elective for Medical Students: In-Person Lectures Versus Inverted Classroom Model [file sj-docx-1-mde-10.1177_23821205261422886.docx]

**Comparison of teaching methods in a Culinary Medicine elective for medical students: In-person lectures vs. inverted classroom model**

Appendix 1: Oxalic acids in different foods.


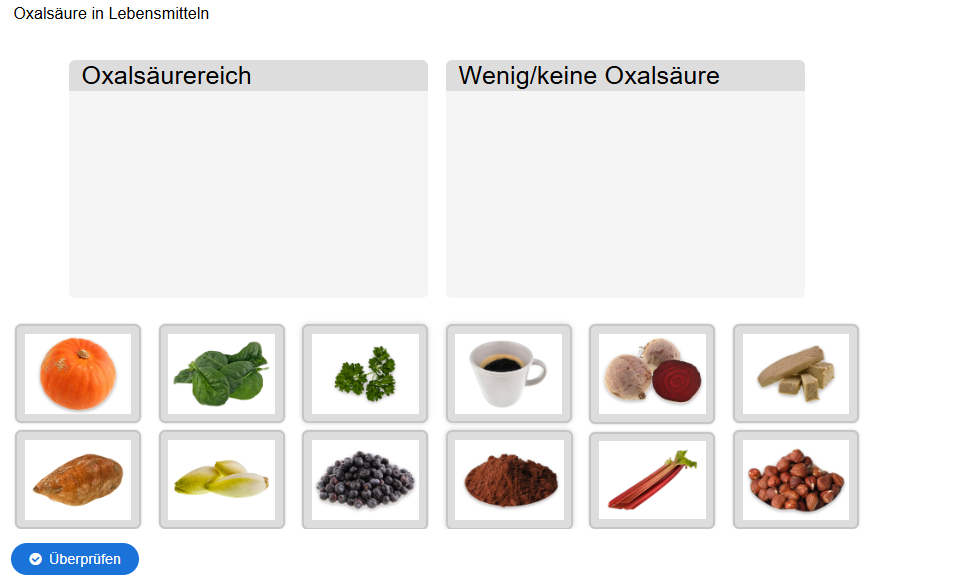


*oxalate-poor*

 **oxalsäurereich** → *high in oxalic acid* oder *oxalate-rich*

*oxalate-rich*

 **oxalsäurereich** → *high in oxalic acid* oder *oxalate-rich*

Appendix 2: Video-tutorial: Oils -Which Oil for what?


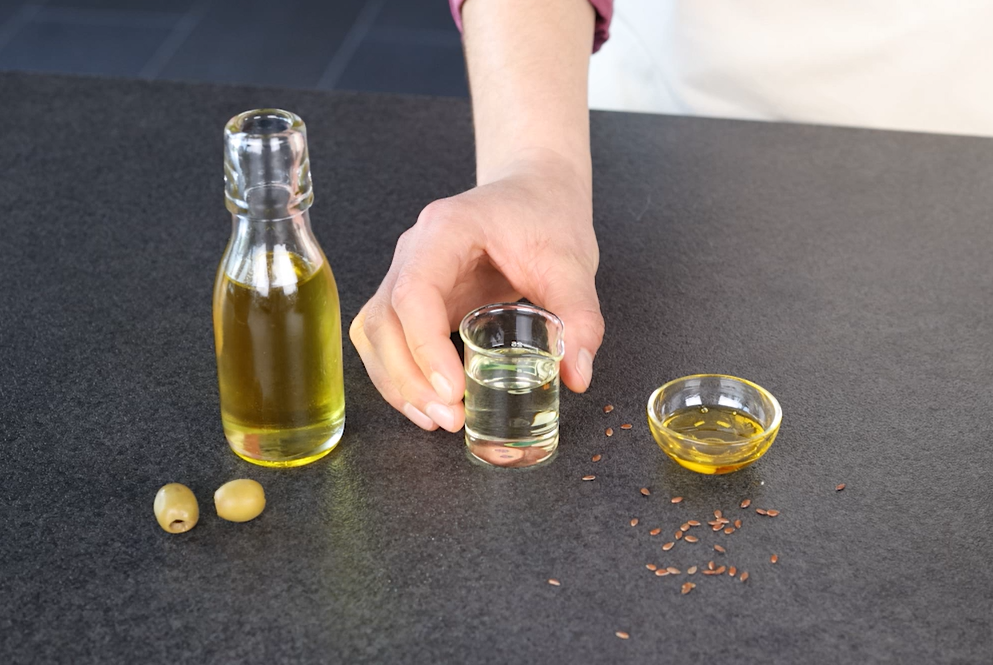


**Table S1.** Counselling competencies paired t-test.

| **Topic** | **T-Value, Inverted Classroom** | **Degree of Freedom, Inverted Classroom** | **Significance (p) (2-Tailed), Inverted Classroom** | **Cohen’s d, Inverted Classroom** | **T-Value, In-Person** | **Degree of Freedom, In-Person** | | **Significance (p) (2-Tailed), Inverted Classroom** | **Cohen’s d, Inverted Classroom** |
| --- | --- | --- | --- | --- | --- | --- | --- | --- | --- |
| Mediterranean Diet | -6.38 | 66 | <0.001 | 1.09 | 9,6 | | 79 | <0.001 | 1,04 |
| Nutrition Arterial Hypertension | -7.34 | 66 | <0.001 | 1.22 | -8,44 | | 79 | <0.001 | 1,16 |
| Vegetarian Diet | -6.01 | 66 | <0.001 | 1.18 | -7,45 | | 79 | <0.001 | 0,96 |
| Low-Fat Diet | -7.11 | 66 | <0.001 | 0.96 | -6,67 | | 79 | <0.001 | 1,02 |
| High-Protein Diet | -6.88 | 66 | <0.001 | 1.08 | -7,56 | | 79 | <0.001 | 1,12 |
| Serving Size | -5.70 | 66 | <0.001 | 1.16 | -8,71 | | 79 | <0.001 | 1,07 |
| Moderate Alcohol | -3.89 | 66 | <0.001 | 1.26 | -6,57 | | 79 | <0.001 | 1,003 |
| Eating Disorders | -4.03 | 66 | <0.001 | 1.09 | -5,97 | | 79 | <0.001 | 1,048 |
| Cholesterol | -5.57 | 66 | <0.001 | 1.18 | -6,54 | | 79 | <0.001 | 1,025 |
| Diabetes Diet | -6.50 | 66 | <0.001 | 1.41 | -9,18 | | 79 | <0.001 | 1,14 |
| Diabetes Weight Loss | -6.07 | 66 | <0.001 | 1.23 | -8,39 | | 79 | <0.001 | 1,14 |
| Obesity Weight Loss | -6.06 | 66 | <0.001 | 1.33 | -8,75 | | 79 | <0.001 | 1,14 |
| Omega Fats ω-3 and -6 | -7.26 | 66 | <0.001 | 1.25 | -10,23 | | 79 | <0.001 | 1,26 |
| Dietary Fats | -6.75 | 66 | <0.001 | 1.14 | -8,61 | | 79 | <0.001 | 1,08 |
| Antioxidants | -7.82 | 66 | <0.001 | 1.09 | -7,08 | | 79 | <0.001 | 1,22 |
| Calories | -5.62 | 66 | <0.001 | 1.41 | -7,77 | | 79 | <0.001 | 1,2 |
| Hydration | -5.97 | 66 | <0.001 | 1.10 | -5,54 | | 79 | <0.001 | 1,1 |
| Celiac Disease | -8.65 | 66 | <0.001 | 1.20 | -11,39 | | 79 | <0.001 | 1,33 |
| Food Allergies | -8.91 | 66 | <0.001 | 1.32 | -12,38 | | 79 | <0.001 | 1,2 |
| Glycaemic Index | -6.93 | 66 | <0.001 | 1.30 | -10,53 | | 79 | <0.001 | 1,2 |
| Fibre | -5.83 | 66 | <0.001 | 1.24 | -10,47 | | 79 | <0.001 | 1,18 |
| Food Label | -6.05 | 66 | <0.001 | 1.43 | -9,78 | | 79 | <0.001 | 1,25 |
| Osteoporosis | -6.52 | 66 | <0.001 | 1.37 | -9,82 | | 79 | <0.001 | 1,32 |
| BMI | -5.80 | 66 | <0.001 | 1.07 | -8,67 | | 79 | <0.001 | 0,98 |
| Aerobic Exercise | -4.03 | 66 | <0.001 | 1.18 | -5,94 | | 79 | <0.001 | 1,09 |

**Table S2.** Counselling competencies unpaired *t*-test.

|  | **T-Value (*t*)** | **Degrees of Freedom (df)** | **Significance (*p*) (2-Tailed)** | **Cohen`s d** |
| --- | --- | --- | --- | --- |
| Mediterranean Diet | 1.55 | 145 | 0.123 | 1.07 |
| Nutrition Arterial Hypertension | -0.05 | 145 | 0.958 | 1.19 |
| Vegetarian Diet | 0.37 | 145 | 0.710 | 1.07 |
| Low-Fat Diet | 0.07 | 145 | 0.947 | 0.98 |
| High-Protein Diet | -0.22 | 145 | 0.829 | 1.11 |
| Serving Size | -1.32 | 145 | 0.188 | 1.12 |
| Moderate Alcohol | -0.75 | 145 | 0.452 | 1.13 |
| Eating Disorders | -0.92 | 145 | 0.359 | 1.07 |
| Cholesterol | 0.31 | 145 | 0.759 | 1.10 |
| Diabetes Diet | -0.26 | 145 | 0.792 | 1.27 |
| Diabetes Weight Loss | -0.84 | 145 | 0.402 | 1.18 |
| Obesity Weight Loss | -1.02 | 145 | 0.310 | 1.28 |
| Omega Fats ω-3 and -6 | -0.69 | 145 | 0.489 | 1.16 |
| Dietary Fats | -1.20 | 145 | 0.233 | 1.18 |
| Antioxidants | 0.50 | 145 | 0.620 | 1.15 |
| Calories | 0.04 | 145 | 0.971 | 1.26 |
| Hydration | 0.65 | 145 | 0.519 | 1.11 |
| Celiac Disease | -2.04 | 145 | 0.043 | 1.28 |
| Food Allergies | -1.16 | 145 | 0.248 | 1.26 |
| Glycaemic Index | -1.54 | 145 | 0.125 | 1.25 |
| Fibre | -2.53 | 145 | 0.012 | 1.21 |
| Food Label | -1.66 | 145 | 0.100 | 1.33 |
| Osteoporosis | -1.62 | 145 | 0.107 | 1.34 |
| BMI | -1.11 | 145 | 0.267 | 1.02 |
| Aerobic Exercise | -0.76 | 145 | 0.448 | 1.13 |

**Table S3.** Attitudes towards nutrition counselling in medical practice paired *t*-test.

| **Question** | **T-Value, Inverted Classroom** | **Degree of Freedom, Inverted Classroom** | **Significance (*p*) (2-Tailed), Inverted Classroom** | **Cohen’s d, Inverted Classroom** | **T-Value, In-Person** | **Degree of Freedom, In-Person** | **Significance (*p*) (2-Tailed), In-Person** | **Cohen’s d, In-Person** |
| --- | --- | --- | --- | --- | --- | --- | --- | --- |
| Nutrition counselling should be routine | -0.76 | 66 | <0.001 | 0.80 | -2.13 | 79 | 0.036 | 0.94 |
| Specific counselling can improve patients’ diet | -1.21 | 66 | 0.002 | 0.80 | -2.83 | 79 | 0.006 | 0.71 |
| Physicians’ counselling can improve patients’ diet | 0.74 | 66 | <0.001 | 0.82 | -4.22 | 79 | <0.001 | 0.76 |

**Table S4.** Attitudes towards nutrition counselling in medical practice unpaired *t*-test.

| **Question** | **T-Value (*t*)** | **Degrees of Freedom (df)** | **Significance (*p*) (2-Tailed)** | **Cohen`s d** |
| --- | --- | --- | --- | --- |
| Nutrition counselling should be routine | -1.03 | 145 | 0.304 | 0.88 |
| Specific counselling can improve patients’ diet | -0.84 | 145 | 0.401 | 0.75 |
| Physicians’ counselling can improve patients’ diet | -3.33 | 145 | 0.001 | 0.79 |

**Table S5.** Nutrition Knowledge paired *t*-test.

| **Question** | **T-Value, Inverted Classroom** | **Degrees of Freedom (df), Inverted Classroom** | **Significance (*p*) (2-Tailed), Inverted Classroom** | **Cohen’s d, Inverted Classroom** | **T-Value, In-Person** | **Degrees of Freedom (df), In-Person** | **Significance (*p*) (2-Tailed), Inverted Classroom** | **Cohen’s d, Inverted Classroom** |
| --- | --- | --- | --- | --- | --- | --- | --- | --- |
| 1. Recommended diet form | -3.99 | 66 | <0.001 | 0.40 | -3.83 | 79 | <0.001 | 0.49 |
| 2 Carbohydrate percentage | -2.55 | 66 | 0.013 | 0.57 | -3.83 | 79 | 0.001 | 0.59 |
| 3 Salt | -9.62 | 66 | <0.001 | 0.52 | -5.59 | 79 | <0.001 | 0.57 |
| 4. Free sugar | -2.51 | 66 | 0.015 | 0.63 | -3.19 | 79 | 0.002 | 0.56 |
| 5. Recommended protein | -3.37 | 66 | 0.001 | 0.58 | -5.39 | 79 | <0.001 | 0.60 |
| 6. Malnutrition syndrome | -7.12 | 66 | <0.001 | 0.53 | -4.13 | 79 | <0.001 | 0.43 |
| 7. Therapy, obesity | -1.40 | 66 | 0.167 | 0.44 | 0.19 | 79 | 0.002 | 0.49 |
| 8. Gout | 0.39 | 66 | 0.698 | 0.63 | -4.13 | 79 | 0.843 | 0.56 |
| 9. Monosaccharide gout | -1.51 | 65 | 0.135 | 0.49 | -3.14 | 79 | <0.001 | 0.56 |
| 10. Dyslipo-proteinemia | -1.09 | 65 | 0.279 | 0.56 | 0.37 | 79 | 0.708 | 0.59 |
| 11. Cereals for celiac disease | -2.49 | 65 | 0.015 | 0.54 | -2.8 | 79 | 0.006 | 0.59 |
| 12. Chronic kidney disease therapy | -3.06 | 65 | 0.003 | 0.60 | -0.78 | 79 | 0.436 | 0.57 |
| 13. Calcium oxalate stones | -2.94 | 66 | 0.005 | 0.62 | -6.01 | 79 | <0.001 | 0.50 |
| 14. Fructose malabsorption | -5.51 | 66 | <0.001 | 0.58 | -2.7 | 79 | 0.008 | 0.53 |
| 15. Omega-3 fatty acid | -3.23 | 66 | 0.002 | 0.61 | -4.13 | 79 | <0.001 | 0.56 |
| 16. Calcium and Vitamin D | 0.00 | 66 | 1.0 | 0.60 | -0.35 | 79 | 0.726 | 0.63 |

**Table S6.** Nutrition Knowledge unpaired *t*-test.

| **Question** | **T-Value** | **Degree o Freedom** | **Significance (p) (2-Tailed)** | **Cohen`s d** |
| --- | --- | --- | --- | --- |
| 1. Recommended diet form | 0.25 | 145 | 0.806 | 0.45 |
| 2 Carbohydrate percentage | -0.47 | 145 | 0.637 | 0.59 |
| 3 Salt | 2.72 | 145 | 0.007 | 0.55 |
| 4. Free sugar | -0.06 | 145 | 0.952 | 0.59 |
| 5. Recommended protein | -1.26 | 145 | 0.208 | 0.59 |
| 6. Malnutrition syndrome | 3.30 | 145 | 0.001 | 0.48 |
| 7. Therapy, obesity | 1.03 | 145 | 0.303 | 0.51 |
| 8. Gout | -2.96 | 145 | 0.004 | 0.60 |
| 9. Monosaccharide gout | -1.03 | 144 | 0.307 | 0.49 |
| 10. Dyslipo-proteinemia | 0.53 | 144 | 0.600 | 0.58 |
| 11. Cereals for celiac disease | -0.22 | 144 | 0.827 | 0.57 |
| 12. Chronic kidney disease therapy | 1.82 | 144 | 0.071 | 0.59 |
| 13. Calcium oxalate stones | -1.22 | 145 | 0.223 | 0.56 |
| 14. Fructose malabsorption | 2.45 | 145 | 0.015 | 0.56 |
| 15. Omega-3 fatty acid | -0.24 | 145 | 0.807 | 0.59 |
| 16. Calcium and Vitamin D | -0.24 | 145 | 0.808 | 0.62 |
