# Supplementary material for: Comparison of Teaching Methods in a Culinary Medicine Elective for Medical Students: In-Person Lectures Versus Inverted Classroom Model
Source: J Med Educ Curric Dev. 2026 Mar 7;13:23821205261422886. doi: 10.1177/23821205261422886 (PMC12967350; doi:10.1177/23821205261422886)
Supplement: sj-pdf-3-mde-10.1177_23821205261422886 - Supplemental material for Comparison of Teaching Methods in a Culinary Medicine Elective for Medical Students: In-Person Lectures Versus Inverted Classroom Model [file sj-pdf-3-mde-10.1177_23821205261422886.pdf]

Culinary Medicine Multicentre Teaching Evaluation from Summer Semester 2022 onwards

Dear students

We have prepared a course-accompanying survey for you to complete at two points in time**: T1: Before the start of the course** and **T2: After the end of the course**. The survey will help us to evaluate and optimise this new, innovative teaching format in nutritional medicine so that future participants can always be offered a highly up-to-date course programme.

ViThank you for your willingness to participate in the **teaching evaluation** of this elective course. This survey is **voluntary, anonymous** and has **no** influence on your grade or passing this module. We also have no access to your personal data such as your name, address or student ID number, and we do not collect any personal or medical data. Once you have completed both surveys (pre- and post-survey) under your **own pseudonym**, the pseudonym will be replaced by a random number. You can terminate this survey at any time without giving reasons and without penalty.

Please answer the questions independently and without using any aids such as the Internet, notes or other people. This is the only way we can get a good picture of the strengths and weaknesses of the module and develop it further.

First, generate a personal 10-digit code according to the following pattern:
Fields 1 and 2 are for the location of the course. Fields 3 and 4 correspond to the second letter of your first name and surname, fields 5 and 6 to the day of your birthday, fields 7 and 8 to the initials of your mother's name. The last two fields, 9 and 10, correspond to the first two letters of your place of birth.

Example: Course in GÖtti ngenFrie da von Dan nen, *03.12.1996;Wil ma von Dannen, Munich

GÖ-	R	A	0	3	W	D	M	Ü

PLEASE GENERATE YOUR CODE HERE

(ONLY FOR THE PAPER QUESTIONNAIRE):

This survey contains 38 questions.

Survey organisation

The following information will be used for later evaluation by cohort

Your personal code:

First, please generate a personal 10-digit code according to the following pattern: Fields 1 and 2 are for the location of the course (= university city, vehicle registration number). Fields 3 and 4 correspond to the second letter of your first name and surname, fields 5 and 6 to the day of your birthday, fields 7 and 8 to the initials of your mother's name. The last two fields, 9 and 10, correspond to the first two letters of your place of birth.

Example: Course in Göttingen (=university town, vehicle registration code) Frieda von Dannen, born 03/12/1996; Wilma Dannen, Munich

GÖRA03WDMÜ

Please enter your answer here:

IMPORTANT: Please make sure you follow the instructions carefully when creating the code so that you receive the same code at both survey times (the instructions will be provided in both surveys). Otherwise, we will not be able to merge the data and will not be able to use your valuable information.

Here is the example again: Course in Göttingen (= university city, vehicle registration number) Frieda Dannen, born 03.12.1996; Wilma Dannen, Munich

GÖ-	R	A	0	3	W	D	M	Ü

Which university are you currently studying at?

BN for UKB Bonn
BB for MHB Brandenburg GI
for UMG-GI Giessen
GÖ for UMG-GAU Göttingen (UMG)

Please enter your answer here:

Please indicate the city of your university in the text. Which university are you currently attending?

BN for UKB Bonn
BB for MHB Brandenburg GI
for UMG-GI Giessen
GÖ for UMG-GAU Göttingen (UMG)

Survey date

Please select one of the following answers:
Please select only one of the following answers:

Preliminary survey (before course

start) Follow-up survey (after

course end)

Special survey (without assignment to course start/end)

Follow-up

Please enter the date of your first day of the Teaching Kitchen course.

Please enter a date:

Enter the date in the format: DD.MM.YYYY

What is your course number or course name according to the course catalogue? For Göttingen, the following applies: W1O9a or W19Ob or W19Oc or W19Od *

Please enter your answer here:

Please enter the short name of your course here.

For Göttingen: W109a or W190b or W190c or W190d

In what format will the course take place?

Please select one of the following answers:
Please select only one of the following answers:

The course takes place in a teaching kitchen in person as an inverted classroom with a preceding ILIAS module The

course takes place in a teaching kitchen in person in a traditional format with a preceding in-person seminar

The course will take place online, interactively with participants cooking themselves

The course will take place as a hybrid event, partly in person in a teaching kitchen and partly online, interactively with participants cooking themselves

Please indicate here whether the course will be held in person, online (interactive with cooking) or hybrid (partly in person/partly online).

Have you ever taken a cooking class before?

Select all applicable options

Please select all applicable answers:

- YES, at school
- YES, at the University of Göttingen
- YES, at another university outside Göttingen
- YES, at another location (e.g. adult education centre, club, health insurance provider, commercial provider, etc.) NO, I have never taken a cooking class

Please indicate your current semester (year)

Please select one of the following answers:

Please select only one of the following answers:

Winter semester 2023/2024

Summer semester 2024 Winter

semester 2024/2025 Summer

semester 2025 Winter semester

2025/2026

Which semester of your studies (since starting your studies) are you currently in? ”

Please select one of the following answers:
Please select only one of the following answers:

- ○ 1
- ○ 2
- ○ 3
- ○ 4
- ○ 5
- 6
- 7
- 8
- 9
- 10
- 11
- 12
- >12

Please list all semesters since starting your studies (for medical courses, list pre-clinical and clinical semesters together)

Please indicate your gender. *

Please select one of the following answers:
Please select only one of the following answers:

- w
- m
- f
- No

How old are you?
Please enter your year of birth *

Only an integer value may be entered in this field. Please enter your answer here:

Is my year of birth

Please enter your year of birth.

Did you complete any training before starting your studies? If so, what kind? ”

Comment if you select an answer

Please select the applicable points and write a comment:

- ☐ YES, vocational training prior to studying medicine
- ☐ YES, academic training prior to studying medicine
- ☐ NO, no training prior to studying medicine

Questionnaire on nutritional medicine counselling practice – students

I cook for myself

Please select one of the following answers:
Please select only one of the following answers:

1x per month/never 1-2 x per week 3-5 x per week 6-7 x per week Daily/more often

I eat (all occasions, at home and away from home) ... *

Please select the appropriate answer for each item:

	1x per month/never	1-2 times per week	3-5 times per week	6-7 times per week	Daily/more often
Vegetables (e.g. carrots, spinach or tomatoes)	○	○	○	○	○
Legumes (e.g. beans, peas or lentils)	○	○	○	○	○
Fruit (e.g. oranges, apples or bananas)	○	○	○	○	○
Nuts or nut butter (e.g. peanuts, almonds, walnuts or cashews)	○	○	○	○	○
Cheese or dairy products (e.g. yoghurt)	○	○	○	○	○
Red and processed meat (e.g. sausage, ham, cutlets, kebabs, hamburgers, steak, hot dogs, etc.)	○	○	○	○	○
Non-fried fish or seafood (e.g. canned,	○	○	○	○	○

	1x per month/never	1-2 times per week	3-5 times per week	6-7 times per week	Daily/more often
Baked or grilled)					
Whole grain products (e.g. whole grain bread, whole grain pasta, oats, brown rice)	○	○	○	○	○
Vegetable oils such as rapeseed oil, sunflower oil and olive oil	○	○	○	○	○
One serving of alcohol (equivalent to 0.331 beer/0.1 wine/2-4cl spirits)	○	○	○	○	○
Sweet pastries (e.g. biscuits, muffins, cakes, doughnuts)	○	○	○	○	○
High-calorie drinks (e.g. lemonade, cola drinks, fruit juice drinks, energy drinks)	○	○	○	○	○
Butter, coconut oil, whole milk products	○	○	○	○	○

My personal opinion on nutritional counselling in medical practice is...

Please select the appropriate answer for each point:

	Strongly disagree	Somewhat disagree	Partly/partly	Agree agree	Agree completely
Advice on nutritional issues should be part of every medical consultation, just like therapy and diagnosis.	○	○	○	○	○
Specific recommendations for changing eating habits can help patients improve their diet.	○	○	○	○	○
Doctors can influence their patients' eating habits if they take the time to discuss the problem with them.	○	○	○	○	○

I am confident that I can advise my patients on the following topics... ”

Please select the appropriate answer for each item:

	Strongly disagree	Somewhat disagree	Partly/partly	Agree Agree	Agree completely
Mediterranean diet and its health benefits	○	○	○	○	○
The right diet for arterial hypertension	○	○	○	○	○
Vegetarian diet and its health benefits	○	○	○	○	○
Low-fat diet and its health benefits	○	○	○	○	○
Protein-rich diet and its health benefits	○	○	○	○	○
Advice on proper portion sizes	○	○	○	○	○
Definition of moderate alcohol consumption and its health effects	○	○	○	○	○

	I don't agree at all	Somewhat disagree	Partly/partly	Agree somewhat	Agree completely
Recognising warning signs and symptoms in patients with eating disorders	◯	◯	◯	◯	◯
The role of (Dietary) cholesterol and saturated fatty acids on blood lipids	◯	◯	◯	◯	◯
Dietary recommendations for type 2 diabetes	◯	◯	◯	◯	◯
The importance of appropriate weight loss for type 2 diabetes	◯	◯	◯	◯	◯
Weight loss strategies for obesity	◯	◯	◯	◯	◯
The role of omega-3 and omega-6 fatty acids in cardiovascular health and examples of foods containing these fatty acids	◯	◯	◯	◯	◯
The role of different dietary fats (saturated, etc.) and relevant dietary examples	◯	◯	◯	◯	◯
Recognising foods rich in antioxidants	◯	◯	◯	◯	◯

	I don't agree at all	Somewhat disagree	Partly/partly	Agree somewhat	Agree completely
The specific calorific value of proteins, carbohydrates and fats and their role in basic metabolic processes	◯	◯	◯	◯	◯
The role of fluid intake in health and fluid requirements adapted to activity and age	◯	◯	◯	◯	◯
Coeliac disease and management strategies for nutrition and everyday life of patients	◯	◯	◯	◯	◯
Food malabsorption and management strategies for nutrition and everyday life of patients	◯	◯	◯	◯	◯
The role of the glycaemic index and glycaemic load in dietary treatment	◯	◯	◯	◯	◯
Fibre in disease prevention and examples of foods containing fibre	◯	◯	◯	◯	◯

	Strongly disagree	Somewhat disagree	Partly/partly	Agree somewhat	Agree completely
Assessment of total calories, saturated fat and sodium content using the nutritional information or ingredient list	◯	◯	◯	◯	◯
Osteoporosis with prevention/treatment strategies for patients' diet and everyday life	◯	◯	◯	◯	◯
Determination of BMI (body mass index) and waist-to-hip ratio (gender-specific) or abdominal circumference	◯	◯	◯	◯	◯
The overall benefit (effect) of aerobic physical activity on health and well-being	◯	◯	◯	◯	◯

Which diet is generally NOT recommended in the Guidelines for Nutritional Medicine in Clinics and Practices (LEKuP)?

Please select one of the following answers:
Please select only one of the following answers:

Vegan diet

Vegetarian diet

Mediterranean

diet

Whole foods according to DGE

recommendations Consistency-modified

whole foods

What should be the proportion of carbohydrates in the total energy intake in a balanced mixed diet according to the DGE (recommendation for healthy people)?

Please select one of the following answers:
Please select only one of the following answers:

- 10
- 20
- 30
- >50
- >60%

What is the maximum recommended total amount of table salt per day according to the DGE (recommendation for healthy people)?

Please select one of the following answers:
Please select only one of the following answers:

- 2.3
- 3.5
- 6.0
- 10 g
- 17 g

What percentage of free sugars (percentage of total energy) is acceptable according to the DGE?

Please select one of the following answers:
Please select only one of the following answers:

- Up to 5 EN%
- Up to 10 EN%
- Up to 15 EN%
- Up to 20 EN%
- Up to 40 EN%

By how much g/kg body weight/day should protein intake be increased in cases of severe malnutrition?

Please select one of the following answers:
Please select only one of the following answers:

up to 0.5 g

up to 0.8 g

up to 2.0 g

up to 3.4 g

up to 4.5 g

Which syndrome can occur during the treatment of severely malnourished individuals?

Please select one of the following answers:
Please select only one of the following answers:

Refeeding syndrome

Waisting syndrome

Metabolic syndrome Short

bowel syndrome Brugada

syndrome

Based on the principles of a balanced diet, which measure is NOT recommended for the treatment of obesity? Select all applicable

options

Please select all applicable answers:

- [ ] An energy deficit of 500-600 kcal/day Reduced fat
- [ ] intake
- [ ] Preference for foods with high energy density Water-rich foods
- [ ] Protein intake as in a whole food diet or t
- [ ]

What is the least concerning aspect of nutrition for gout/hyperuricaemia?

Please select one of the following answers:

Please select only one of the following answers:

Sweets Non-

alcoholic beer

Carbonated mineral water with lemon

Lemonade

Offal

Which monosaccharide is particularly problematic in cases of elevated uric acid levels?

Please select one of the following answers:
Please select only one of the following answers:

Glucose

Fructose

Galactose

All monosaccharides are equally problematic Monosaccharides have no effect

on uric acid levels

Which dietary measure is NOT recommended for hypertriglyceridaemia-type dyslipoproteinemia?

Please select one of the following answers:
Please select only one of the following answers:

Normalisation of body weight

Reduction of monounsaturated fatty acid intake to less than 10% of total energy intake Increase in

polyunsaturated fatty acid intake to more than 10% of total energy intake Limitation of monosaccharide and

disaccharide intake

Increase intake of soluble fibre

Which type of grain can still be consumed in cases of manifest coeliac disease? *

Please select one of the following answers:
Please select only one of the following answers:

- Whole wheat
- Barley
- Spelt
- Millet
- Ancient grains (e.g. einkorn, emmer)

What measures should be taken for chronic kidney disease depending on the stage?

Please select one of the following answers:

Please select only one of the following answers:

Protein restriction in early stages General

calorie deficit

Increased phosphate consumption (e.g. through increased cheese consumption)

Vitamin A supplementation in all stages

Potassium-rich diet in all stages (e.g. through increased consumption of legumes)

Which foods should be avoided if you have calcium oxalate stones? ? *

Please select one of the following answers:
Please select only one of the following answers:

Rhubarb

Chocolate

Nuts Spinach

Egg

What improves fructose tolerance in patients with fructose malabsorption?

Please select one of the following answers:
Please select only one of the following answers:

Addition of salt

Simultaneous intake of glucose

Consuming fructose-containing foods in isolation Simultaneous

intake of unsaturated fatty acids Simultaneous intake of lactose

Which of these oils contains only small amounts of omega-3 fatty acids?

Please select one of the following answers:
Please select only one of the following answers:

Rapeseed

oil Walnut

oil Linseed

oil

Sunflower oil

Fish oil from fatty sea fish

Which conditions do not constitute an exception to the recommended calcium intake of 1000 mg/day and vitamin D intake of 800 IU/day?

Please select one of the following answers:
Please select only one of the following answers:

Primary hyperparathyroidism Glucocorticoid-induced

osteoporosis Calcium oxalate stones

Hypercalcaemia

Active granulomatous diseases

Questions about well-being (WHO 5)

To conclude the survey, here are five final questions about your well-being

In the last two weeks...

Please select the appropriate answer for each item:

	All the time	Most of the time	A little more than half the time	A little less than half the time	Occasionally	Never
...I felt happy and in a good mood	○	○	○	○	○	○
...I felt calm and relaxed	○	○	○	○	○	○
...I felt energetic and active	○	○	○	○	○	○
...did I feel fresh and rested when I woke up	○	○	○	○	○	○
...my everyday life was full of things that interested me	○	○	○	○	○	○

Feedback

ViThank you for participating in the elective course!

To conclude, we would appreciate your feedback.

Please tell us briefly about your experiences in the course.

- What did you like, what didn't you like?
- What could be added?

Please only answer this question if the following conditions are met:
The answer was NOT 'Preliminary survey (before course start)' for question ' [PRE00002]' (survey date) Please enter your

answer here:

Thank you very much for taking the time to give us your feedback at the end of the course. This helps us to continuously improve our course offerings and incorporate your suggestions.

How would you rate the course? (from 1 very good to 5 poor)

Please only answer this question if the following conditions are met:
The answer was NOT 'Preliminary survey (before course start)' for question ' [PRE00002]' (survey date)

Please select one of the following answers:
Please select only one of the following answers:

1 Very good

2 satisfactory

3 satisfactory

4 sufficient

5 poor

How satisfied were you with the teachers?

Please answer this question only if the following conditions are met:
The answer was NOT 'Preliminary survey (before course start)' to question ' [PRE00002]' (survey date)

Please select one of the following answers:
Please select only one of the following answers:

- Very satisfied
- Satisfied
- Dissatisfied
- Not at all satisfied

The course was interesting for me and relevant to current issues

Please only answer this question if the following conditions are met:
The answer was NOT 'Preliminary survey (before the start of the course)' for question ' [PRE00002]' (survey date)

Please select one of the following answers:
Please select only one of the following answers:

Strongly disagree

Disagree Somewhat

agree Somewhat disagree

Agree somewhat

Agree completely

I would recommend the course

Please only answer this question if the following conditions are met:
Answer was NOT 'Preliminary survey (before course start)' for question ' [PRE00002]' (survey date)

Please select one of the following answers:
Please select only one of the following answers:

Strongly disagree

Disagree Partly

agree/Partly disagree

Agree Agree

Thank you for participating in the survey!

If you would like to provide us with brief feedback in keywords, please send us an email at kursmanager@culinarymedicine.de
WiWe look forward to receiving your suggestions and criticism.

Your Culinary Medicine Team

21 September 2023 — 4:46 p.m.

Submit your survey.

Thank you for completing the questionnaire.
